# Socioeconomic Deprivation and Opioid Consumption: An Analysis Across England

**DOI:** 10.3390/ijerph22050750

**Published:** 2025-05-09

**Authors:** Sara Appleby, Othman Al Musaimi

**Affiliations:** 1 School of Pharmacy, Newcastle University, Newcastle upon Tyne NE1 7RU, UK; 2 Department of Chemical Engineering, Imperial College London, London SW7 2AZ, UK

**Keywords:** opioid, deprivation, socioeconomic factors, addiction, prescription

## Abstract

Concerns sparked by the US opioid epidemic have led to increased attempts to reduce England’s overall opioid consumption. Variations in health literacy across the country have led to differing prescribing practices, with increased chronic conditions appearing in areas of increased socioeconomic deprivation. This review investigated the relationship between increased opioid utilization and increased socioeconomic deprivation, aiming to highlight areas of England which have the highest opioid consumption. The review has investigated a range of socioeconomic factors, such as unemployment and fuel poverty, which have consequently influenced the higher frequency of opioid prescribing in areas where these factors were highest. Opioid abuse was most prevalent in areas with higher levels of deprivation. Geographically, areas with the highest levels of deprivation, and consequent opioid consumption, were situated in the North of England, with cities like London having a significantly lower consumption rate in comparison. These trends are a good starting point when designing future opioid epidemic-prevention strategies, as specific areas can be targeted to propel a reduction in opioid prescribing and addiction and thus decrease the likelihood of an opioid crisis forming. Although a longitudinal study would have strengthened the findings of this review, it was not feasible due to practical constraints.

## 1. Introduction

**Figure 1:**
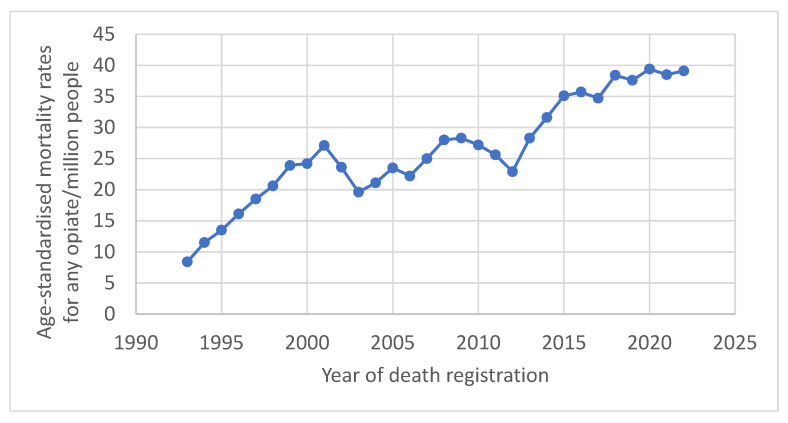
Annual opioid-related mortalities across England. Data compiled from the Office of National Statistics (ONS).

The frequency of the prescription of opioids in primary care across England has steadily decreased since 2016, following a 34% boom in prescriptions nationwide between the years 1998 and 2016 [1]. This drop in opioid prescriptions in recent years is the result of an increase in knowledge surrounding the harms associated with these drugs and subsequent stricter prescribing guidelines. Despite an attempt to control the usage of prescription opioids, opioid-related mortalities have continued to rise annually, regardless of efforts put in place to reduce these figures; these trends are displayed in Figure 1 [2].

Data such as these imply that the concerning problem of opioid abuse continues to thrive across England, stressing how susceptible England is to the formation of an opioid epidemic, like the one currently in the US. The US has battled with an ongoing opioid epidemic since the 1990s, after Purdue Pharma’s (Stamford, CT, USA) fraudulent marketing of OxyContin was a major contributor to the opioid epidemic in the US. Subsequent waves of heroin and synthetic opioid use in 2010 and 2013, respectively, further fueled the epidemic; this crisis continues to grow to this day, contributing to around 45 deaths in the US per day in 2021 alone [3]. Interestingly, US drug overdose deaths have dropped by 24% in the past year, with about 87,000 fatalities from October 2023 to September 2024—the lowest since June 2020, according to new CDC data [4]. The devastating impacts on the US’s overall public health caused by this epidemic serve as a warning to England and stress the need to avoid the same fate; increased homelessness and unemployment are just two factors that are directly negatively impacted by this problem in the US [5]. Minimizing opioid dependence and mortalities is crucial for England in order to prevent a similar outcome, as the ever-growing epidemic continues to wreak havoc across the US despite beginning over three decades ago [3].

Changes to prescribing practices in England in recent years is a key strategy which has been implemented to aid in reducing the number of opioid prescriptions dispensed. The addictive nature of these drugs carries the risk of developing tolerance and dependence when they are used for long periods of time, consequently making them discouraged in the management of chronic conditions [6,7,8]. Despite this knowledge, around 50% of patients who were prescribed an opioid in 2017 had a repeat prescription continuously fulfilled for at least a 1-year period, implying that a sizeable proportion of patients are maintained on long-term opioid therapy regardless of the current guidance [6,9]. Furthermore, the existing literature has proven the significant risk between persistent opioid use and subsequent opioid-related deaths, with persistent opioid users being 2.6 times more likely to die from opioid-related deaths than a non-chronic user [10]. These facts stress the importance of the role prescribers across England play in preventing an opioid crisis, as prescribers create a barrier between patients and the accessibility of these drugs. The rates of tolerance, dependence, and deaths caused by opioids can therefore be reduced and even avoided altogether by the intervention of healthcare professionals through prescribing practices and patient education.

Health education aimed at the general public has also increased in recent years to help reduce opioid use, as the degree to which an individual understands their general health directly impacts their quality of life. Health literacy is defined by the Centers for Disease Control and Prevention (CDC) as “the ability of an individual to find, understand and use information and services to inform health-related decisions and actions for themselves and others”, with limited health literacy increasing the likelihood of an individual choosing poorer lifestyle behaviors [11]. Areas in England with a higher incidence of lower health literacy contain a higher proportion of individuals who chose to smoke, excessively consume alcohol and partake in illicit drug use within their communities [11]. Consequently, there is an increased risk of developing chronic conditions and premature death in less health-literate populations in comparison to more health-literate ones, further reinforcing the importance of education in general health and well-being. Financial deprivation is a factor seen to have a strong link to levels of health literacy [12]. Individuals living in areas of financial deprivation are twice as likely to have a low health literacy than those in the least deprived areas, indicating communities of a lower socioeconomic status in England generally suffer from more healthcare issues than those of a higher socioeconomic status [13]. Financially deprived communities also contain increased levels of crime, unemployment and homelessness; factors which all negatively impact an individual’s health literacy [14,15]. Furthermore, a low socioeconomic deprivation status and low health literacy significantly increase the risk of drug misuse, further complicating health conditions already existing in these populations [16].

In this review, we will investigate the relationship between socioeconomic deprivation and opioid consumption across England as a whole. This review subsequently aims to highlight any geographical variations within opioid prescribing that are possibly linked to socioeconomic differences. This topic is being investigated as more socioeconomically deprived areas are more likely to have complicated health needs, due to lower health literacy, which may require treatment with opioids and increase the frequency of prescribing in these locations. Moreover, prescription opioids can be a gateway to opioid dependence, as previously discussed, and so investigating populations with the highest levels of prescribing can aid in designing and implementing future interventions. Overall, this review seeks to support the reduction in England’s overall opioid consumption by highlighting factors which influence high levels of opioid prescribing and illicit opioid use, aiding in the prevention of an opioid epidemic forming in England, such as the one seen in the US.

## 2. Study Selection

The studies included in this review must focus on opioid utilization and prescribing patterns in England, particularly examining the relationship between opioid prescribing and socioeconomic deprivation indicators such as unemployment, fuel poverty, income levels, and education. The research should provide a geographical analysis of opioid consumption across different regions of England, with an emphasis on regional disparities, such as higher consumption in the North compared to the South. Eligible studies include observational studies, cohort studies, cross-sectional studies, systematic reviews, and meta-analyses published in peer-reviewed journals, government reports, or public health studies within the last 15 years to ensure relevance. Exclusion criteria include studies not published in English, research focusing on opioid consumption outside of England, or studies that do not provide England-specific data. Additionally, studies primarily investigating opioid use for recreational purposes rather than medical prescribing patterns, those lacking socioeconomic analysis, single-case studies, or research with small sample sizes that do not provide meaningful statistical insights will be excluded. Furthermore, outdated studies conducted before the last 15 years will not be considered unless they offer historically relevant insights into trends in opioid prescribing and socioeconomic deprivation.

## 3. Governmental Funding

Government funding controls the level of wealth of each local authority across England, with the amount of funds allocated to each differing between regions. As a result, there can be differing levels of wealth and deprivation scattered geographically. Areas in which lower funds are allocated to their local government often have increased levels of deprivation, which can be demonstrated by the impacts budget cuts have on these areas. According to a report conducted by the Joseph Rowntree Foundation between 2010 and 2014, cuts to finances have been seen to disproportionately target already-deprived areas across England, particularly lowering the funds for social care in these areas [17]. A second study conducted by Alexiou and co-workers demonstrates the impact of financial cuts to housing services, which can increase homelessness and promote a reduced quality of life [18]. These two examples show how low government funding can accelerate the deprivation of an area and the people who live there. Increased deprivation has been linked to poorer health literacy and an increased incidence of chronic diseases, which, as this study will go on to demonstrate, are both factors in increased opioid prescribing and usage.

As previously mentioned, financial cuts have been linked with exacerbating the socioeconomic deprivation of an area and its inhabitants. An observational study conducted by Friebel and co-workers investigated the impacts of austerity measures on the rates of opioid-related hospitalizations and opioid-related mortalities between 2010 and 2017 [19]. Austerity measures were implemented in 2010 by the newly elected Conservative government with the aim of lowering national debt, resulting in reduced funding being received by local authorities across a range of sectors [19]. Financial expenditure records were accessed through publicly available Government documents, with this study specifically investigating changes to spending within the social care and housing sectors [19,20]. Opioid-related hospitalization data were obtained from the Hospital Episode Statistical Database, with opioid-related mortality data collected from the ONS [19]. The study concluded that areas situated in the Midlands, the North West and the South West of England experienced the highest financial reduction in social care and housing funding throughout the study period [19]. These regions maintained heightened unemployment levels, averaging around 7.9% above the national average, despite a country-wide decrease at this time [19]. These results imply that reducing the amount of funds available to an area creates the risk of higher unemployment levels, which can consequently trigger a cascade of detrimental socioeconomic-associated issues [19]. A demonstration of this is the significant relationship discovered between increased opioid abuse (opioid-related harm and mortalities combined) and increased unemployment levels, as the areas which experienced the highest levels of opioid abuse were the North West, the South West, and the South East of England [19]. In summary, areas hit the hardest by financial cuts experienced heightened unemployment levels as well as heightened opioid abuse, and were mainly concentrated in the North West and South West of England [19]. Changes in Government spending across England could be proposed as a risk factor for increased opioid abuse due to the discovery of this relationship, and subsequent future public health campaigns could be implemented to help reduce overall opioid use.

The time period of 7 years was a substantial time to conduct such a large-scale study, with a wide range of data being collected to accurately represent financial and opioid use variations across England within this time. Also, the inclusion of both opioid-related hospitalizations and opioid-related mortality data allows for a more accurate representation of the overall extent opioid abuse across England than if one data set was used alone. There was no differentiation between prescription or illicit opioids within this study; thus, it is unknown which indication made up a higher proportion of hospitalizations and deaths. It is harder to implement specific interventions if the indication of the opioids in use is unknown. These data only include recorded issues associated with opioids and so do not include opioid use which does not lead to hospitalization or death, suggesting that this study may not fully capture the true extent of opioid use in the area.

Additional literature supporting a positive relationship between financial deprivation and opioid usage across England includes a study completed by Vandoros et al. [21]. The number of opioid prescriptions dispensed between 2011 and 2017 was obtained from the GP prescribing database for each practice across England [21]. Each practice was subsequently linked with fuel poverty percentages and median incomes for the area in which it resided, allowing a comparison to be made between deprivation and opioid prescribing; fuel poverty is defined as spending 10% or more of a household’s income on heating a home [21,22]. Furthermore, the effects of unemployment and opioid prescribing were also investigated, with data obtained from Normis [21]. The study concluded that fuel poverty is linked to increased opioid prescribing. Unemployment also significantly increases the rate of opioid prescribing, which is higher in areas of increased deprivation [21].

Inclusion of fuel poverty as a determinant of deprivation can be used to explain why opioid prescriptions are higher in these areas, as more chronic conditions are associated with colder homes and are hence treated with opioids [22]. This study demonstrates how the physical effects of deprivation can directly cause an increase in opioid prescriptions through directly causing physical harm, posing a risk of opioid dependence and addiction in these populations.

The study period and data set were substantial and accurately represented prescription opioid use across England. However, one weakness of this study is that there was no mention of geographical regions, only “areas” of higher or lower deprivation, making it harder to compare this study geographically to other literature. This study also includes the use of prescription opioids in opioid-agonist therapy used to treat addiction, which is of note as it is the only study reviewed in this paper to do so. Further research could investigate geographical and socioeconomic variation across England in the frequency of opioid-agonist therapy, due to the lack of literature in this topic. Whilst this study’s main focus was the influence of unemployment on opioid prescribing, it was included in this review to demonstrate how different factors of deprivation, such as fuel poverty, can similarly influence opioid prescribing. This study demonstrates how employment may have a protective role over an individual’s health and consequent opioid consumption.

## 4. Prescribing Patterns Across England

Continuing to focus on opioid utilization within primary care, Chen et al. investigated opioid prescribing patterns in relation to socioeconomic factors, as well as geographical prescribing patterns across England in 2015 [23]. Prescribing data were obtained from NHS digital for 7856 practices across England, with each practice being assigned a deprivation score depending on the postcode they reside in—the Index of Multiple Deprivation (IMD) [23]. The IMD is the official measure of deprivation within small areas of England, with a smaller score indicating a higher level of deprivation [24]. Each practice was grouped into their respective clinical commissioning group (CCG) to allow for an average number of opioid prescriptions to be obtained and compared [23]. London, Birmingham, Manchester, and Newcastle were chosen to specifically investigate differences in prescribing based on geographical location [23]. It is important to note that the opioid prescribing rates were estimated and calculated based on standardized defined daily doses and each practice’s registrants.

**Figure 2:**
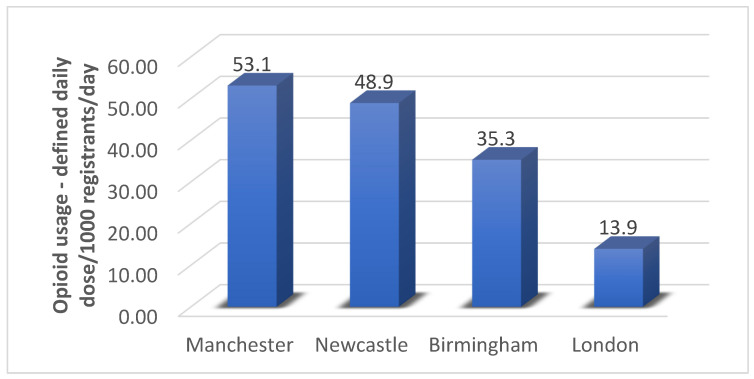
The difference in estimated opioid utilization across the four cities investigated within the subgroup investigation. A significant increase in opioid utilization by 1.0 DDD/1000 registrants/day (95% confidence interval: 0.89, 1.2; *p* < 0.001) was observed.

This cross-sectional study demonstrated variation in opioid utilization between the 209 CCGs investigated, with the highest prescribing rates existing in the northern and eastern regions of the country. A higher number of opioid prescriptions were dispensed in areas with lower IMD scores, indicating a positive relationship between socioeconomic deprivation and opioid utilization. With respect to the four cities compared, the highest number of opioid prescriptions were dispensed in Manchester, with London having the fewest prescriptions (Figure 2).

**Figure 3:**
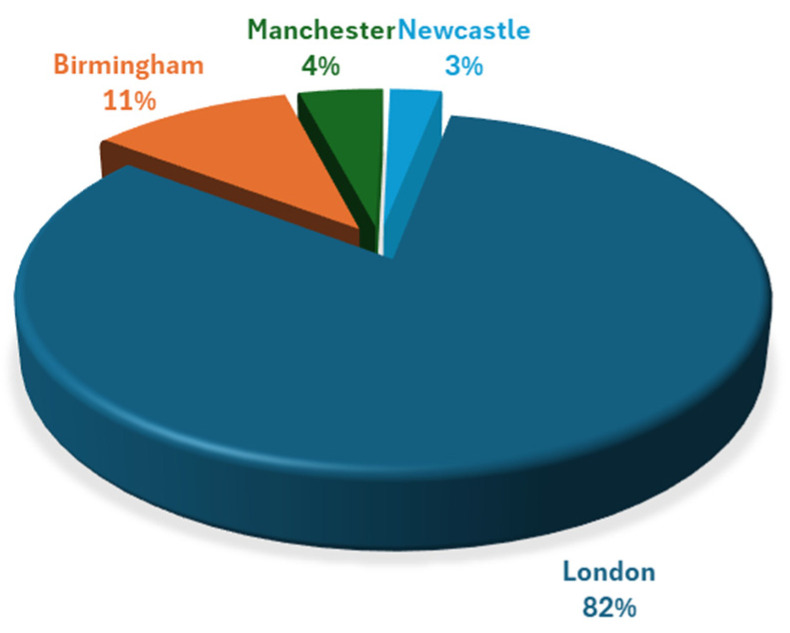
Population sizes of cities across England. Data obtained from the 2011 census from the ONS (36–71).

Manchester, Birmingham, and London all displayed significant correlations between increased opioid prescribing and increased deprivation, whereas Newcastle showed no significant correlation. The lack of correlation observed in Newcastle could be explained by the fact this city is more deprived overall in comparison to the other three investigated [25]. The austerity measures described previously in this review provide an explanation as to why Newcastle, being one of the most northern cities, has more deprived areas in comparison to the other cities [19]. The overall decrease in funding across Newcastle could cause similar IMD levels city wide, thus not providing a large enough range in scores to show a significant relationship between differing scores and prescribing. Revisiting the effect of austerity measures in respect to this study is important as they came into effect before this study period, meaning the results of this study were directly impacted by the financial cuts of 2010. Austerity measures also explain why Manchester, saw the highest opioid prescriptions in this study, due to higher financial cuts and a northern location equating to more opioid prescriptions in comparison to cities situated in the south; the number of prescriptions in Manchester could be higher than in more-northern Newcastle due to having a higher population [26]. Figure 3 demonstrates population sizes of cities across England, where it is shown Newcastle has a lower population than Manchester according to the 2011 census [27].

The inclusion of this study in this review allows for geographical comparisons between opioid prescribing and socioeconomic deprivation, showing how differences in deprivation influence prescribing practices across different regions of the country. The inclusion of country-wide data for both opioid prescriptions and IMD scores allowed for an accurate representation of geographical diversity within both sectors, thus allowing for the relationships between both data sets to be distinguished. The four cities chosen varied in location, population size, and demographics, providing an insight into factors which potentially influence increased opioid utilization.

This study, however, only took place over a one-year period, which is the shortest study period included in this review, especially short for an ever-changing financial situation across England. This study could have also looked further into more than just four cities in more depth, which would more accurately show the north–south prescribing gradient already present in the four cities investigated.

It is important to note these opioid prescription rates are estimates for each practice involved in this study and are not exact measures of each individual’s prescriptions. As a consequence, the number of prescriptions may not reflect the true extent of opioid prescribing at a practice level and instead just reflects the number of patients per practice. Future studies should be performed measuring true numbers of opioid prescriptions at practice levels for true comparison between geographical areas. This study was included in this review to reflect the estimated variation in opioid prescribing between different geographical regions across England, with respect to the four cities highlighted as case studies.

### 4.1. Prescribing Patterns in Liverpool

Chen et al. and Begley et al. conducted a study focusing on opioid prescribing and deprivation variations across Liverpool to observe diversity in this city alone [23,28]. This observational study was conducted between 2016 and 2018, with prescribing data obtained from GP surgeries situated within Liverpool CCG; 62 out of 88 practices complied and released their data [28]. IMD scores were once again used to measure deprivation across Liverpool, specifically the north, center, and south of the city [28]. Details of opioid prescriptions were collected from each practice for each patient.

**Figure 4:**
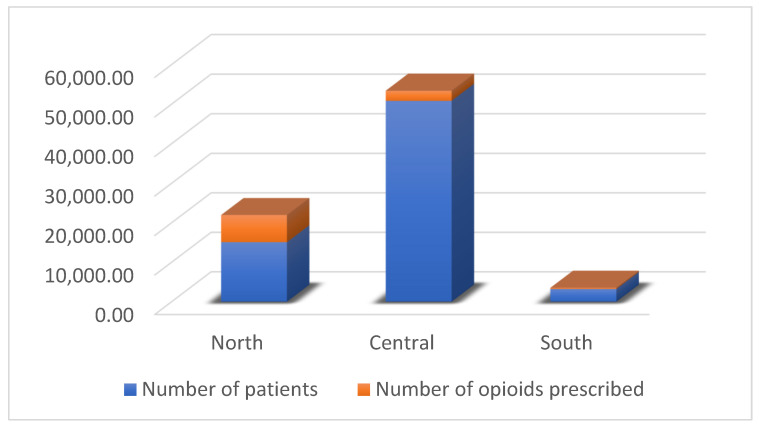
Opioid prescribing frequency in comparison to the number of patients registered in each area of Liverpool. Percentages are calculated by dividing the total number of patients prescribed any opioid by the total number of registered patients in a GP locality. The percentage of patients on multiple opioids is calculated similarly. The proportion of patients prescribed more than one opioid is determined as follows: (patients prescribed > 1 opioid/patients prescribed 1 opioid) × 100.

The study concluded that the north of Liverpool was the most deprived area, having the highest incidence of opioid prescriptions and an increased likelihood of patients receiving more than one opioid prescription compared to the rest of the city [28]. On the contrary, the south of Liverpool saw the lowest number of prescribed opioids and was the least deprived area, demonstrating the correlation between opioid utilization and deprivation [28]. This relationship is further reinforced by the trends in opioid prescribing in comparison to how many patients reside in an area (Figure 4).

The data show the dramatic difference in how many patients reside in the center of Liverpool in comparison to the north; however, the north of Liverpool has over double the number of prescriptions compared to that in the center, with the only other variable being the IMD score. This proves the reoccurring trend of higher incidences of opioid prescribing in areas with higher deprivation. The results from this study further strengthen the view that opioid usage is overall increased in areas of increased socioeconomic deprivation. This study provides a more in-depth look at the impacts of deprivation variation across a city, prompting curiosity as to why opioids are in fact utilized to a higher degree in these areas. This study provides a good template for other research to be conducted in other cities, allowing for a nationwide comparison of opioid prescribing at the city level.

It is important to note that not all 88 GP practices within the LCCG participated, and so these results may not accurately depict the full scope of opioid usage across Liverpool; it is unknown which areas of Liverpool the remaining 26 surgeries resided in. Thus, this stresses the importance of primary care organizations involvement in studies such as this one, as the results can be used to increase public health and education and be tailored to the areas that need them. The study period of two years is a relatively short timeframe compared to others in this review, but is not the shortest and so obtained good results for the smaller size of the study. Furthermore, every opioid prescription was included in the totals, even when patients had more than one prescription. This could mean the ratio between opioid prescriptions to patients across the areas of Liverpool may not be accurate, as some patients are prescribed multiple opioid items.

### 4.2. Private Prescribing Patterns Across England

Steering away from public healthcare, Martus et al. investigated changes within the frequency of private opioid prescriptions, and how prescribing practices differed geographically within the private healthcare sector [29]. Their retrospective observational study was conducted between the years 2014 and 2021, utilizing data from the NHS Business Services Authority (BSA) [29]. The private opioid prescription data were mapped out geographically to allow for comparisons across England [29].

The results showed that North London had the highest incidence of private opioid prescriptions, dispensing 5313 items and accounting for 74.6% of all opioids dispensed across the study period [29]. There were lower private opioid prescriptions dispensed towards the North of England, with Yorkshire having the lowest amount prescribed, accounting for only 0.69% [29]. Overall opioid consumption decreased across the study period, following trends of an overall reduced opioid consumption across England in recent years [29].

This study’s author’s conclusions and results differed to those of the previous studies included in this review, as the south of the country, especially London, has consistently had some of the lowest opioid prescribing rates geographically in every study previously reviewed. The opposing results from this study corresponding to private opioid prescriptions mirror the fact that London is the wealthiest city in England; therefore, a larger proportion of individuals in this city can afford such costs to access private healthcare. The variation in private opioid prescriptions between the North and South of England mirrors the socioeconomic gradient discussed previously, with more individuals in the South having the money and resources available to have increased health literacy, health resources, and consequently, better public health [30]. The inclusion of this study in this review allows for a different perspective on opioid prescribing, as only 10.6% of the English population are covered by private healthcare insurance [31]. Consequently, the results of this study are reflective of a wealthier proportion of the population, as the high costs of private healthcare are less likely to be accessible to individuals living in deprivation [32].

An advantage of this study is that it shows that most private healthcare is concentrated in London, thus reinforcing the trends previously described that there is less money available in the North of England [19]. The study period of seven years is a substantial time to conduct an observational study, especially one that covers the whole country, thus providing a large data set for analysis.

The limitations of this study are the lack of doses and indications for what private opioids were prescribed for, which could be useful information for future opioid public health interventions. Only Schedule 2 and 3 opioids were investigated, excluding codeine preparations, which are Schedule 5, unlike any other piece of literature in this review [33]. As codeine is one of the most prescribed opioids in England, the exclusion of codeine from this study may not accurately depict private opioid prescribing, and repeating the study including codeine may alter the results observed [34]. This study further strengthens the north–south financial and health divide and shows how individuals living in London can access better healthcare and have improved health literacy in comparison to areas of deprivation.

Most of the studies were longitudinal or cross-sectional in nature, allowing for different ranges of time to be investigated, with both being observational study techniques. The different time periods covered across all of these studies allow for a good representation of opioid prescribing trends throughout recent years to be shown, with the most recent study utilizing data up to 2021 and the oldest dating to 2010.

## 5. Conclusions

All the studies, apart from the study conducted by Vandoros and co-workers, excluded the indication of opioids in opioid-agonist therapy, thus removing data for drugs such as methadone and buprenorphine as this is their main indication [21]. The inclusion of these data could possibly have changed the results across these studies, but does, however, leave room for further study on geographical and socioeconomic variations in opioid-agonist therapy. Moreover, preparations such as co-codamol; codeine with paracetamol (BAN); or codeine with acetaminophen (USAN) are available OTC and subsequently were not monitored across all studies. It is possible that the scope of opioid usage across different geographical and socioeconomic regions was not properly represented due to a lack of OTC monitoring. Further investigations could be carried out into the number of OTC opioids purchased in different regions across England; however, it is understood that this is a difficult factor to measure due to the increased availability of this preparation. Only one of the studies included, by Friebel et al., showed the effects of overall opioid abuse and gave an insight into use of illicit opioid use, as there was a lack of research into illicit opioid use across England when researching for this review [19]. This consequently allows for further research to be carried out into variations in illicit opioid use across England; however, it is again noted that this is more difficult to investigate in comparison to prescription opioids. The most recent study available on topics associated with the aim of this review utilized data from up to 2021, meaning that more recent data should begin to be collected on opioid prescribing variations and socioeconomic deprivation now to show more recent and up-to-date trends.

The literature compiled in this review has continuously proven that there are significant relationships between increased opioid consumption, both prescription and illicit, and increased levels of socioeconomic deprivation. The North of England was consistently observed to have higher rates of opioid prescribing, as well as reduced finances and employment rates. Geographical areas with the lowest financial hardships were seen to have reduced opioid prescriptions and opioid abuse. Factors such as fuel poverty, unemployment, and austerity measures were investigated to allow for a range in different socioeconomic factors to be investigated, which all had a positive relationship with increasing opioid utilization.

The studies in this review overall imply that there is a reduced health literacy and an increase in chronic conditions in areas of increased socioeconomic deprivation, which in turn explain the geographical variation in opioid utilization. The relationships discovered in this review act as a starting point for future public health interventions to be implemented, such as increased personalized medicine reviews in areas of lower social status, to aid in reducing the number of patients taking opioids long term. Whilst a longitudinal study would have confirmed the outcome of this review, this was not feasible due to practical constraints. It was not possible for us to collect individual patient data or carry out subsequent follow-ups on opioid usage, as well as socioeconomic situation, country wide and compile the results in a study. As a result, an analysis of the existing literature led us to create this review and come to these conclusions. This review reveals opportunities for research into possible genetic risk factors for addiction and whether they are limited to deprived communities, helping to aid in the formalization of prevention strategies [35]. In addition, the ratio of prescription to illicit opioid use would also be beneficial to understanding how most individuals develop dependence, again giving an insight into how to manage this issue. This, in turn, helps steer England away from the possibility of an opioid epidemic forming, avoiding the same fate as the US.
